# Pickering Emulsions Stabilized with Curcumin-Based Solid Dispersion Particles as Mayonnaise-like Food Sauce Alternatives

**DOI:** 10.3390/molecules27041250

**Published:** 2022-02-12

**Authors:** Larissa C. Ghirro, Stephany Rezende, Andreia S. Ribeiro, Nuno Rodrigues, Márcio Carocho, José Alberto Pereira, Lillian Barros, Bogdan Demczuk, Maria-Filomena Barreiro, Arantzazu Santamaria-Echart

**Affiliations:** 1 Centro de Investigação de Montanha (CIMO), Instituto Politécnico de Bragança, Campus de Santa Apolónia, 5300-253 Braganca, Portugal; larissa.ghirro@ipb.pt (L.C.G.); rezendes@ipb.pt (S.R.); nunorodrigues@ipb.pt (N.R.); mcarocho@ipb.pt (M.C.); jpereira@ipb.pt (J.A.P.); lillian@ipb.pt (L.B.); 2 Campus Campo Mourão, Universidade Tecnológica Federal do Paraná (UTFPR), P.O. Box 271, Campo Mourao 87301-899, Brazil; bdjunior@gmail.com; 3 Laboratory of Separation and Reaction Engineering-Laboratory of Catalysis and Materials (LSRE-LCM), Faculdade de Engenharia, Universidade do Porto, Rua Dr. Roberto Frias, 4200-465 Porto, Portugal; asribeiro@fe.up.pt

**Keywords:** solid dispersions, Pickering emulsions, natural-based formulations, k-carrageenan, curcumin, low-fat foods

## Abstract

Pickering emulsions, which are emulsions stabilized by colloidal particles, are being increasingly positioned as novel strategies to develop innovative food product solutions. In this context, the present work aims to develop Pickering emulsions stabilized by natural-based curcumin-loaded particles produced by the solid dispersion technique as promising mayonnaise-like food sauce alternatives. Two particle formulations (KC1 and KC2) were produced using k-carrageenan as the matrix material and different curcumin contents, then employed in the preparation of three Pickering emulsion formulations comprising different oil fractions (φ) and particle concentrations (KC1 φ 0.4 (4.7%), KC2 φ 0.4 (4.7%) and KC2 φ 0.6 (4.0%)). The creaming index tests accompanied by the optical microscopy analysis evidenced the good stability of the developed products for the tested period of 28 days. The final products were tested concerning color attributes, pH, oxidative stability, textural, and nutritional composition, and compared with two commercial mayonnaises (traditional and light products). Overall, the produced emulsions were characterized by a bright yellow color (an appealing attribute for consumers), an acidic pH (similar to mayonnaise), and a considerably improved oxidative stability, implying a foreseeable longer shelf life. The sauce KC1 φ 0.4 (4.7%) showed a similar texture to the light commercial mayonnaise, being a promising alternative to conventional sauces, holding a low-fat content and potentially added benefits due to the curcumin and virgin olive oil intrinsic properties.

## 1. Introduction

The food industry is increasingly focused on developing novel products able to satisfy actual consumption trends. Among them, healthy, fat-reduced and bioactive-based functional products should be highlighted. In this context, Pickering emulsions are promising systems able to provide a solution to some of these challenges.

Pickering emulsions are mixtures of two immiscible liquids (typically oil and water), being one (dispersed or internal phase) dispersed in the form of droplets into the second phase (continuous or external phase). They are stabilized by the adsorption of solid colloidal particles at the interface, forming a rigid physical barrier to decrease the Gibbs free energy, ensuring high stable emulsions [1,2]. The stabilization by Pickering particles promotes greater stability to coalescence and Ostwald ripening when compared to conventional emulsions that use chemical emulsifier molecules [3].

The effective action of the particles as Pickering stabilizers is associated with some of their particular characteristics, including an appropriate wettability for both phases, specific particle sizes, and concentration in the emulsion formulation. The wettability, usually determined by contact angle (θ) measurements, evidences the relative affinity of the particles for the oil and water phases [1,4], being the preferable range comprised between 30 and 150°. Generally, hydrophilic particles (θ < 90°) form oil-in-water (O/W) emulsions while hydrophobic particles (θ > 90°) lead to water-in-oil (W/O) emulsions [5]. The particle size influences the droplet size of the emulsion, rheology, stability and even the sensorial perception in food applications, with smaller particles holding a greater interface coverage capacity, enabling the stabilization of a higher emulsion volume when compared to larger particles [6]. Similarly, a higher content of particles avails the stabilization of a larger interfacial area, resulting in finer emulsions [7], even though not a guarantee of the adsorption of all particles at the interface [4,8]. Their excess, located in the continuous phase, or the use of high internal oil volume fractions, then so-called High Internal Phase emulsions, favors the formation of highly stable viscous systems, including gels [8]. These systems stand out as attractive structures for several industrial applications [9]. Among them, novel foods, e.g., sauces mimicking sensorial properties of conventional ones.

The potential of Pickering emulsions for the food sector results from the appealing nutritional, and textural properties, as well as from their potential role as carriers of bioactive compounds, conferring functionality to the final formulations. Among the various production methods for particle formation, solid dispersion is a promising and innovative technique. It is typically used in the pharmaceutical area [10], being recently extended to the food industry, namely to increase the hydrophilicity of curcumin as a natural colorant [11]. The process involves the incorporation of a hydrophobic compound (e.g., curcumin) into a hydrophilic carrier matrix, promoting water dissolution, bioavailability and bioaccessibility. In a final step, the formed dispersion is dried, spray-drying being an attractive technique to obtain the final solid particles. Amid many advantages of using this technique for particle production, we can mention its simplicity, low cost, and high efficiency, providing the formation of small and uniform particles [12], which is a relevant parameter for the Pickering dispersions development.

Regarding the nature of the Pickering stabilizers, increasing attention is focused on the use of natural compounds and the inclusion of functionalities. Among bioactives, curcumin, a natural polyphenolic compound found in the turmeric herb rhizome, has recently gained considerable interest due to its health-promoting properties, such as anti-inflammatory, anticarcinogenic, and antioxidant characteristics [13]. However, overcoming its low bioaccessibility is a major challenge due to its hydrophobicity, low oral bioavailability, in addition to its tendency to degrade in the intestinal tract [14]. The selection of the k-carrageenan, a natural polymer, as a carrier is an attractive strategy to produce natural-based solid dispersion particles [11]. Moreover, k-carrageenan holds a gelling character, which is an important characteristic for the textural properties of sauces commonly used in the food industry, thus guarantying conformity with food products.

The importance of producing natural-based particles to use as Pickering stabilizers, as well as their potential to develop novel all-natural food products, is being highlighted in many recent studies. Nevertheless, the developed systems often lack the stability and physicochemical properties of commercial products, which emphasizes the need to proceed with research in the field [15,16]. In this context, the production of foods based on natural ingredients, free of allergens and with enhanced properties, is positioned as a promising alternative to traditional products. Namely, the actual healthy lifestyle trends, which include fat-reduced and vegan consumption habits, imply the need to develop alternatives to enlarge the offered product range. According to recent projections, the global market of vegan sauces was rated as USD 194.6 million, with an expected annual growth rate of 8.2% until 2027 [17]. Concurrently, the Food Allergy Research and Education (FARE) estimated that in 2019, 32 million US citizens suffered from food allergies (e.g., eggs or fish), pointing out the interest in developing novel vegan products as a solution to overcome these needs [17].

In this work, Pickering emulsions stabilized with curcumin-based solid dispersion particles were produced and proposed as potential alternatives to conventional mayonnaise-like sauces. For that, two solid dispersion formulations based on k-carrageenan and curcumin were produced and tested in the preparation of Pickering emulsions seeking stable products with textural properties similar to the ones of the target products. The particles were characterized in terms of color attributes, wettability and particle size. The prepared emulsions were analyzed concerning stability, colorimetry, pH, oxidative stability, and textural properties, in comparison with two reference foods, namely commercial mayonnaises (traditional and light). Moreover, the advantages of the newly developed products in terms of nutritional value were discussed. Overall, apart from the approach offered by Pickering emulsions to develop innovative products, to the best of our knowledge, the use of particles produced by the solid dispersion technique as Pickering stabilizers was never reported. These particles have the advantage of easily introducing hydrophobic compounds, such as curcumin, in their composition, which in combination with the hydrophilic polymer, offer the possibility to tune the wettability of the particles. Moreover, the combination of curcumin, a compound with recognized bioactivity and color attributes, with the natural polymer k-carrageenan, offers the advantage of producing all-natural-based products with wellbeing benefits and color attributes.

## 2. Results and Discussion

In this work, two Pickering stabilizers were produced using the solid dispersion technique. K-carrageenan was used as the carrier polymer, which was loaded with different curcumin contents (15 and 8.9 wt%) to form, respectively, KC1 and KC2 particles. Thereafter, these particles were used in the preparation of three emulsions by varying the particles’ content (PC, 4.0 and 4.7 wt%), and the oil volume fraction (φ of 0.4 and 0.6), being designated as KC1 φ 0.4 (4.7%), KC2 φ 0.4 (4.7%), and KC2 φ 0.6 (4.0%).

### 2.1. Characterization of the Particles

**Figure 1:**
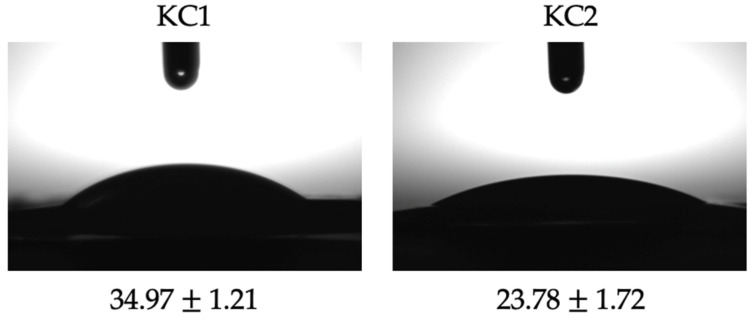
Contact angle for KC1 and KC2 particles produced by the solid dispersion technique.

The obtained solid dispersion particles (KC1 and KC2) were characterized concerning wettability, particle size, and color. The wettability values, evaluated by measuring the water contact angle, are shown in Figure 1. Both formulations showed values in the range comprised between 15° and 90°, evidencing their hydrophilic character, and, consequently, their propensity to stabilize O/W emulsions. The higher values observed for KC1 particles indicate their higher stabilizing role, comparatively with the KC2. It is known that irreversible adsorption of the Pickering particles at the oil and water interface is favored when the contact angle is between 30° and 150°, the range at which the desorption energy of the particles is several orders of magnitude higher than the thermal energy of the Brownian movement [18].

The obtained particle sizes corresponding to 10%, 50%, and 90% of the total particles, in volume and number, respectively, D_10_, D_50_ and D_90_, are summarized in Table 1. It is well known that the main variations occurring in the volume and number results derive from the influence of the larger and smaller particles, respectively, even when they are present at low levels. Summarizing, the particles showed considerable small sizes in volume, with D_50_ values ranging from 2.39 to 3.29 µm, indicating their potential as Pickering stabilizers, as reported by Low et al. [18]; namely, smaller particles promote the stability of Pickering emulsions due to their faster adsorption kinetics, facilitating their packaging at the liquid’s interface. For both samples, a multimodal distribution in volume was observed, resulting from particle agglomeration, an effect often caused by the spray-drying process [19]. The particle size in number showed that the two formulations presented a similar distribution. Their unimodal profile in number is considered an important factor for producing stable Pickering emulsions [20]. The uniformity of the results corroborates the reproducibility of the procedures, for the manufacturing of standardized particles, an essential step for industrial implementation of the process.

The particles’ L*, a* and b* color parameters are shown in Table 2. Curcumin is an important natural compound present in the rhizomes of turmeric (*Curcuma longa*) being widely used as a colorant and flavoring agent in foods, in addition to their antioxidant activity. When properly dispersed in an aqueous medium, it reveals a lemon-yellow color in an acid medium, becoming orange in a basic medium [21]. According to this assumption, the particles resulted in high b* values, which are indicative of their strong yellow tone (considering the b* positive values), supported by the low pH used for their fabrication. The high luminosity L* values indicated that samples were clear. In contrast, the a* values close to 0, particularly for the KC2 sample, revealed no significant effects derived from green and red colors (e.g., hues ranging from −a* to +a* values, respectively).

### 2.2. Evaluation of the Effective Role of KC1 and KC2 as Pickering Stabilizers

**Figure 2:**
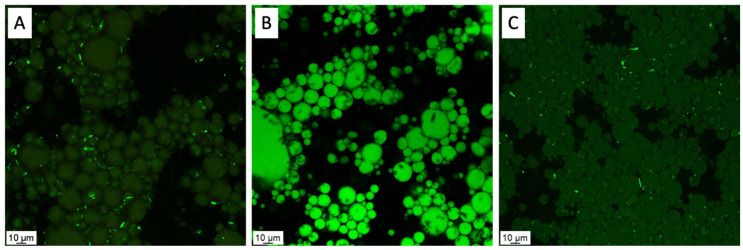
Confocal microscopy images showing the natural fluorescence of curcumin included in the KC1 particles of KC1 φ 0.4 (4.7%) sample (**A**), KC2 particles of KC2 (KC2 φ 0.4 (4.7%) (**C**), and the fluorescence of the oil phase stained with Nile red of the sample KC1 φ 0.4 (4.7%) (**B**).

The effective role of the produced particles (KC1 and KC2) as Pickering stabilizers was evaluated by analyzing the samples KC1 φ 0.4 (4.7%) and KC2 (KC2 φ 0.4 (4.7%) by confocal analysis, and the results are summarized in Figure 2. The confocal analysis was performed taking advantage of the curcumin natural fluorescence (488 nm, green fluorescence), and after being dyed with Nile red to stain the oil phase (488 nm, green fluorescence). The analysis of Figure 2A,C, which reports the natural fluorescence of curcumin included in the stabilizing KC1 and KC2 particles, respectively, corroborate their effective attachment to the dispersed phase, whose oily nature was proved after analyzing the sample KC1 φ 0.4 (4.7%) stained with Nile red (Figure 1B). In this case, the observed Nile red fluorescence is due to both the particles and oil. Summarizing, the confocal analysis corroborated the effective absorption of the particles at the O/W interface as well as the O/W nature of the emulsion. Moreover, no dispersed particles were detected in the continuous phase, reinforcing their tendency to be adsorbed at the O/W interface and thus effectively stabilizing the emulsion.

### 2.3. Emulsion Stability

**Figure 3:**
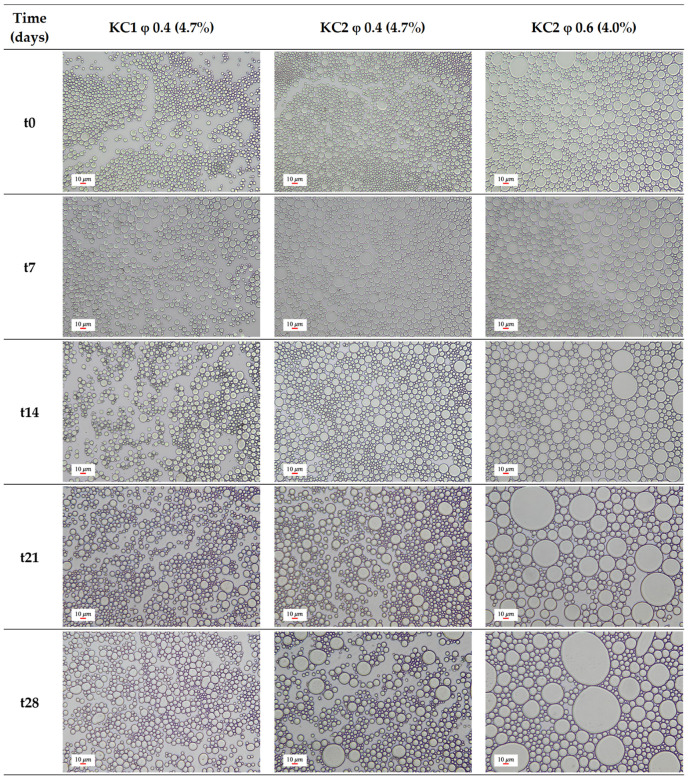
Optical microscopy images (200× magnification) of the three produced Pickering emulsions (KC1 φ 0.4 (4.7%), KC2 φ 0.4 (4.7%), and KC2 φ 0.6 (4.0%)) over time (t0–t28 (days)). Scale bar of 10 μm.

The stability of the Pickering emulsions produced using KC1 and KC2 particles (KC1 φ 0.4 (4.7%), KC2 φ 0.4 (4.7%), and KC2 φ 0.6 (4.0%)) was evaluated every 7 days until a total period of 28 days (0, 7, 14, 21, and 28 days) at room temperature by monitoring the creaming index (CI) and acquiring the corresponding optical microscopy images. The evolution of emulsion morphology with time is shown in Figure 3 and the corresponding average droplet size is summarized in Table 3. It is known that the oil volume fraction and the particles’ concentration are fundamental factors affecting the emulsion stability. Namely, when the oil volume fraction increases, the area to be stabilized also increases, generating larger droplets and less stable emulsions. Similarly, low particles concentration might not be enough to effectively cover all the interfacial area, an effect that tends to be counterbalanced by increasing droplet size and decreasing emulsion stability [22]. This effect might correspond to the case of the KC2 φ 0.6 (4.0%) sample, namely the lower particle content (4.0%) and the high oil volume fraction (φ 0.6) resulted in significantly large droplets. These emulsions showed a more substantial increase in droplet size over time, as observed in Figure 3 and corroborated by the values presented in Table 3, comparatively with emulsions produced with 4.7% particles and a 0.4 oil volume fraction (KC1 φ 0.4 (4.7%), and KC2 φ 0.4 (4.7%)), being the less stable formulation among the three produced ones. These observations are consistent with previously reported studies [23,24]. In conclusion, the systems based on KC1 and KC2 particles produced with φ 0.4 and a particle concentration of 4.7% originated the emulsions with the smaller droplet size, which remained stable throughout the analyzed period, thus indicating a high resistance to physical instabilities, such as coalescence and Ostwald ripening.

**Figure 4:**
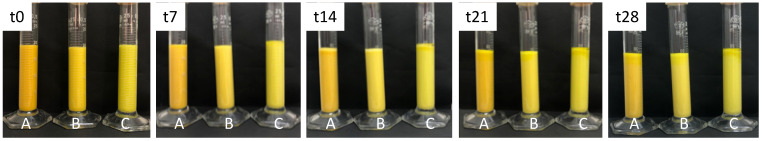
Photographic registration of the cream index evolution along time (0, 7, 14, 21, and 28 days) for the produced emulsions: KC1 φ 0.4 (4.7%) (A), KC2 φ 0.4 (4.7%) (B), and KC2 φ 0.6 (4.0%) (C).

The CI of the emulsions, shown in Figure 4, is a measure of the macroscopic emulsion stability. Considering the lower density of the oil phase, the oil droplets tend to rise to the top of the sample. Oppositely, the aqueous phase tends to accumulate at the bottom when instability occurs [25]. Overall, the obtained results evidenced the macroscopic stability of the three emulsions over the tested period of 28 days (no serum phase was formed and thus CI was 0). However, a slight oil accumulation was observed at the top of the emulsion for the sample KC2 φ 0.6 (4.0%), particularly after 14 days, a fact related to its lower stability, in agreement with the optical microscopy results.

### 2.4. Comparative Study with Commercial Products

Having in view the potential of the developed products to be used as mayonnaise-type food sauces substitutes, the formulations produced using the extra virgin olive oil were compared with reference to commercial products (traditional and light mayonnaise). These formulations were analyzed considering color attributes, pH, oxidative stability, texture, and nutritional value. The color attributes of the produced Pickering emulsions and reference products are summarized in Table 4, together with pH and oxidative stability analysis.

The emulsions showed high luminosity, represented by the high L* values, a strong influence of the yellow color reflected by the highly positive b* values, which can be associated with the remarkable coloring power of curcumin in acidic media, results in accordance with the work reported by Wulandari et al. [26]. The negative values of a* in the KC2 φ 0.4 (4.7%) and KC2 φ 0.6 (4.0%) samples reflect the effect of the green color characteristic of the olive oil, evidencing a higher influence, i.e., higher a* values, in the sample with higher oil content (KC2 φ 0.6 (4.0%)). Additionally, the lower a* values for the formulations produced with the KC2 particles (Table 2), compared with the ones using KC1 particles, can also justify the lower values of the corresponding emulsions. Regarding the mayonnaises, both samples presented low b* values, corresponding to the yellow color and high L* values associated with the luminosity or the white color. The bright yellow color is a strong visual attribute of mayonnaise, being appreciated by consumers in general. Comparing the commercial mayonnaises with the samples, it is noticeable that the samples revealed a stronger yellow color, mainly the samples KC1 φ 0.4 (4.7%) and KC2 φ 0.4 (4.7%), evidencing the curcumin coloring ability conferred by the particles, which might be an appealing attribute for consumers.

Considering the pH analysis, the emulsions showed an acidic character, which was associated with the particle’s acidity themselves. This fact is derived from the used production conditions (KC1 at 4.50 and KC2 at 3.58), evidencing the relevant influence of the particles on the final emulsion properties. To highlight that the acidic pH was also observed for the mayonnaise, being the characteristic responsible for interfering not only in the sensory properties, being also important to ensure microbiological safety, preventing microorganism’s growth and enhance food preservation.

Lipid oxidation is also an essential parameter concerning the deterioration of commercial food emulsions, whose adverse effects impact quality, namely in the sensorial aspects, undesirable rancidity, and generation of potentially toxic compounds. The degradation of nutritional and sensory properties generally occurs due to the formation of primary and secondary compounds, including peroxides, aldehydes and ketones [27].

The Pickering emulsions showed a considerably improved oxidative stability (20–28 h) compared with the analyzed mayonnaises (0.3–0.7 h), indicating higher resistance to the peroxidation by-products, a fact potentiating a longer shelf life for the Pickering emulsions. In fact, Pickering emulsions are recognized to have improved chemical stability compared with conventional emulsions due to the enhanced separation of the pro-oxidants in the aqueous phase from the oil, thanks to the formation of the particle’s thicker physical interfacial layer. In addition, particles produced from compounds with antioxidant activity, such as curcumin, can also enhance the inhibition of pro-oxidants, thereby improving the emulsion oxidative stability [28].

The three produced Pickering emulsions showed quite similar results, namely 27.98 h, 24.28 h and 20.32 h for KC2 φ 0.4 (4.7%), KC1 φ 0.4 (4.7%) and KC2 φ 0.6 (4.0%) formulations, respectively. The decreasing tendency could be related to the higher amount of oil in the emulsions, the main product susceptible to oxidation over time.

The high demand for clean-label products has promoted the search for new alternatives to conventional food additives. Among others, Pickering emulsions are positioned as promising systems to structure safer and healthier foods, besides enabling texture modulation by changing, e.g., the concentration of the Pickering particles [29]. In this context, the textural and sensorial quality, and digestibility of the Pickering emulsion-based foods, have also directed their applicability towards the development of low-fat and reduced-calorie food products [30]. For this purpose, their production from materials with gelling capacity can mimic semisolid textures of trans- or saturated fats [31], ensuring similar textural properties. In this context, the textural properties of the emulsions and the traditional and light mayonnaises were evaluated over time (0 and 10 min) in duplicate, and the results expressed as average ± SD are summarized in Table 5.

The properties determined from the textural analysis provide emulsion physical characteristics. The firmness, defined as the maximum force peak supported by the sample when pressured by the disk, indicates emulsion stiffness. The area under the peak refers to the consistency of the sample (e.g., the higher the value, the thicker the sample is). The cohesiveness, maximum force peak depicted inversely to the firmness, denotes the work needed to overcome the internal emulsion bonds when the disk is pulled out from the sample. This force, represented as a negative to reflect the decompressing of the sample, indicates consistency/resistance to sampling flow on the disk. The area under this peak reflects the work of cohesion [32,33].

It is known that, in general, emulsion systems with smaller droplets result in firmer and more adhesive products [34]. This effect was observed for the obtained emulsions, where the firmness, consistency, cohesiveness and work of cohesion values increased with the droplet size reduction, as supported by the discussed optical images in Section 2.2. KC1 φ 0.4 (4.7%), the sample with the smallest droplet size, showed improved texture attributes, namely in firmness, consistency, cohesiveness and work of cohesion. The performed measurements indicate, for time 0, that there are no statistically significant differences (*p* < 0.05) when comparing this sample with the commercial light mayonnaise for all the analyzed parameters, evidencing their similar textural properties. In addition, for all samples, only slight variations were observed in the texture properties over time (from 0 to 10 min), behavior corroborated by the statistical analysis, where no significant differences were found between the two times (0 and 10 min) for almost all the evaluated parameters. This fact indicated their stability to the mechanical impact occurring during the texturometer tests. The phenomenon can be related to the rigid layers responsible for covering the droplets (intrinsic characteristics of the Pickering emulsions) [35], which reinforce the resistance of the system. Comparing the texture properties of the Pickering emulsions with those of the mayonnaises, similar behaviors were observed, especially in the case of KC1 φ 0.4 (4.7%), showing comparable values to light mayonnaise. Other reported works [32] have analyzed the texture properties of low-fat mayonnaise based on different fat substitutes, showing similar results, namely in what concerns firmness values (between 256.56 and 349.35 g), cohesiveness (120.45 to 198.45 g) and work of cohesion (983.53 and 1685.41 g·s). However, slightly higher consistency values were reported (2785.35–3654.78 g·s).

The produced emulsions showed reduced lipids (fat) content (36.5, 36.5, and 54.7 g/100 g, for KC1 φ 0.4 (4.7%), KC2 φ 0.4 (4.7%), and KC2 φ 0.6 (4.0%), respectively), compared to the traditional mayonnaise (80 g/100 g), but were slightly higher than the light mayonnaise (26.4 g/100 g). However, it is worth noting that the light mayonnaise has a high carbohydrate content (24.5 g of which 8.6 g are sugars), components that are frequently used to counterbalance the reduction of the lipids in these formulations, namely to maintain the textural and sensory properties. Recent studies claimed that a high-carbohydrate diet could be just as harmful as a high-fat diet for the development of illnesses such as liver injury, becoming an increasing concern [36]. In this context, the developed Pickering emulsions were positioned as a potential alternative to replace fat-containing products with low carbohydrate content. Comparing the emulsions, those produced with an oil fraction of 0.4 become more attractive than the ones produced with 0.6. Thus, the KC1 φ 0.4 (4.7%) and KC2 φ 0.4 (4.7%) presented the most promising nutritional balance, corresponding to a lipids value of 36.5 g/100 g, revealing the potential for application in the food market as a fat-reduced mayonnaise substitute.

## 3. Materials and Methods

### 3.1. Materials

Solid particles were prepared using curcumin (65% purity) purchased from Sigma-Aldrich, and k-carrageenan (a natural encapsulating material) supplied from Acros Organics. Tween 80 (Alfa Aesar, Haverhill, MA, USA) and absolute ethanol (Honeywell, Charlotte, NC, USA, 99.8%) were used as surfactant and solvent, respectively. The citric acid (99.5%) and sodium citrate (99.0%), purchased from PanReac, were used to prepare the buffer solutions to control the pH during the particles’ production.

Pickering emulsions were prepared using an extra virgin olive oil produced in the region of Trás-os-Montes and Alto Douro, which was obtained from a local producer. Traditional and light mayonnaise purchased from a local market in Bragança (Portugal) were selected as comparative food matrices.

### 3.2. Preparation of the Particles

The solid particles were produced by the solid dispersion technique according to previous work [11]. This methodology is reported to be an effective method to improve the solubility of poorly water-soluble compounds [37], a limiting factor for bioaccessibility [14]. In this work, the solid dispersion particles were explored as Pickering stabilizers. Briefly, Tween 80 and k-Carrageenan were solubilized in 100 mL of an aqueous buffer solution (citric acid/sodium citrate) at a specific pH (4.5, and 3.58). Simultaneously, curcumin was solubilized in 50 mL of ethanol and poured into the aqueous solution. The mixture was sonicated (model Qsonica, Q500, 500W) at 70% of potency for 10 min with 30/10 s *on/off* cycles to avoid overheating. The dispersion was dried by spray-drying (Mini Spray Dryer B290 Büchi, Flawil, Switzerland) under the following conditions: nitrogen flow at 667 L/h, inlet temperature at 140 °C, outlet temperature in the range of 70 °C, solution flow of 11 mL/min and aspiration rate of 35 m^3^/h. The produced particles were stored at 4 °C, protected from light before characterization and use for Pickering emulsions production. The prepared formulations, selected from the base work [11], and designated as KC1 and KC2, are shown in Table 6.

### 3.3. Preparation of the Emulsions

Pickering emulsions were produced according to previous work with some modifications [38]. Briefly, the particles were dispersed in distilled water. Then, the oil was slowly added using a peristaltic pump at 3500 rpm (ISM596B, Ismatec SA, Opfikon, Switzerland) under constant stirring (stirring plate, VWR, Radnor, PA, USA). The resultant pre-emulsion was subsequently emulsified using an Ultra-Turrax homogeniser (Unidrive X1000 Homogenizer Drive from CAT Scientific, Staufen, Germany) at 13,500 rpm for 7 min. The final emulsions were stored at room temperature.

Three emulsions were prepared by varying the PC with respect to the aqueous phase, and oil volume fraction (φ). The chosen formulations (KC1 φ 0.4 (4.7%), KC2 φ 0.4 (4.7%), and KC2 φ 0.6 (4.0%)), which were fixed based on a previous initial screening (study considering φ of 0.4 and 0.6 with PC of 3, 4, 4.5, 4.7 and 5%), and are summarized in Table 7. Among the screened formulations, the 3 selected ones were the only ones not showing a prompt phase separation after production.

### 3.4. Characterization of the Particles

The particles were characterized concerning wettability, particle size, and color.

The wettability, which characterizes particles’ hydrophilic/hydrophobic character, was evaluated using an optical contact angle device (OCA15 Plus, Dataphysics, Filderstadt, Germany) [38]. For that, pellets were produced by compressing the particles between two smooth glass slides at room temperature. After placing the pellets on the device platform, a droplet of 4 μL of deionized water was deposited with a high-precision injector at 25 °C. The images were acquired using a digital camera attached to the equipment, and the contact angle values obtained by adjusting the profile data to the Laplace–Young equation. The results were obtained by computing four droplets and reported as average ± SD.

The particle size, in volume and number, was determined by laser diffraction technique using a Malvern Mastersizer 3000 equipped with a dispersion Hydro MV (Malvern) unit (Worcestershire, UK cidade, país). Samples were analyzed, averaging 5 measurements at ambient temperature, using distilled water as the dispersing medium. For both volume and number measurements, D_10_, D_50_ and D_90_, were determined to indicate the particle size corresponding to 10%, 50% and 90% of the particles in the sample, respectively [11].

The color analysis was performed using a colorimeter (model CR-400, Konica Monolta Sensing Inc., Osaka, Japan) equipped with a liquid samples cell. The values of L*, a* and b* were determined using a diaphragm aperture of 8 mm, averaging 3 measurements for each sample at ambient temperature. The L* value indicates the sample brightness (black to white spectra reflected by the 0 to 100 range); the a* coordinate represents the influence of green when negative (−a*) and red when positive (+a*); and the b* value shows the variation of blue when negative (−b*) and yellow when positive (+b*) [11].

### 3.5. Characterization of the Emulsions

The Pickering emulsions were characterized concerning their stability to storage, an important attribute for final product development. The effective role of KC1 and KC2 particles as Pickering stabilizers was evaluated by confocal analysis. Moreover, they were compared with chosen model foods (traditional and light mayonnaise) concerning color (the same methodology as described in, pH, oxidative stability, and textural analysis. Nutritional value for the emulsions was estimated taking into consideration their composition (oil, water and particles). For the mayonnaises, the nutritional tables supplied with the commercial product were used.

Confocal microscopy was used to check the effective adsorption of the produced particles (KC1 and KC2) to the O/W interface. For that, the natural fluorescence of curcumin (488 nm, green fluorescence) and of Nile red (488 nm, green fluorescence) used to stain the oil phase (0.1% *w*/*v*) were recorded. The apparatus was a Leica TCS-SP5 AOBS (Leica Microsystems Inc., Heidelberg, Germany). The images were digitally recorded and processed with LasX software.

Stability was accessed by determining the CI (%) across a period of 28 days with sampling points at 0, 7, 14, 21, and 28 days. CI, a stability indicator, was determined according to the method described by Nikolovski et al. [39], with some modifications. Briefly, the prepared emulsions were transferred to 25 mL graduated test tubes, sealed to prevent evaporation, and kept at room temperature during the evaluation period. CI was calculated using Equation (1) where *H_s_* correspond to the height of the aqueous layer (serum layer) and *H_t_* is the total height of the system.

(1) $$CI\;\left(\%\right)=\frac{Hs}{Ht}\times 100$$

In parallel, morphology and the average droplet size were checked by optical microscopy along the test period of 28 days using a Nikon Eclipse 50i optical microscope (Kawasaki, Japan), equipped with a Nikon Digital Sight camera for image acquisition and results in treatment. The images were obtained by putting a droplet of the emulsion in a glass slide covered with a glass lamella. The average droplet size was calculated by averaging the diameter of 30 randomly selected droplets with the results expressed as average ± SD [40].

The pH of the emulsions and of the traditional and light mayonnaises was measured using a pH meter (Inolab pH 720, WTW, Weilheim, Germany) at room temperature. The probe was inserted directly into the analyzed products. The measurements were made in triplicate and reported as average ± SD.

The oxidative stability of the emulsions and the commercial mayonnaise was evaluated in duplicate through a conductivity method using Rancimat 743 equipment (Metrohm, Herisau, Switzerland). Dry, clean, and filtered air was bubbled at a flow rate of 20 L/h through 3 g of the sample at 120 °C. The oxidation compounds, more polar than the triglycerides (including hydroperoxides, alcohols or carbonyl compounds) formed over time, are transported by the airflow to bubbling distilled water, whose conductivity increase is continuously measured. The period between the starting point of the test until the oxidation products formation, that is, the induction period reflects the oxidation stability of the samples. This interval, calculated directly using the software associated with the equipment, was determined between the beginning of the record and the intersection point of the tangents of the curve [41].

The texture profile analysis of the emulsions and the reference mayonnaises were analyzed using a TA-XT plus texture analyzer from Stable Micro Systems (Vienna Court, Godalming, UK), equipped with a 5 kg load cell, according to the method of Roriz et al. [42], with some modifications. Firmness, consistency, cohesiveness and work of cohesion of the samples were determined in compression mode using a P/45 45 mm aluminum probe and a rear extrusion cell of 35 mm diameter compression disc. The samples, covering up to 75% of a cylindrical acrylic container (50 mm diameter and 35 mm height), were analyzed in target mode set to 10 mm of “distance” and trigger mode in “Auto (Force)” corresponding to 5 g. All the samples were analyzed in duplicate at each evaluated time, namely at 0 and after 10 min, and the results expressed as average ± SD, using a pre- and post-test speed of 5 mm/s and test speed of 3 mm/s. The results were obtained using Exponent software version 6.1.11.0, proprietary to Stable Micro Systems. The statistical analysis to identify the significant differences between the samples was carried out using ANOVA and Tukey tests (α = 0.05) performed with Statistica StatSoft, Inc. (2011) (version 10, Tulsa, OK, USA).

The nutritional composition of the emulsions was estimated based on their formulation, assuming the main contribution of the used virgin oil, and the negligible role of the other components (water and the Pickering particles) for the nutritional value. For comparison purposes, the nutritional tables of the reference mayonnaise were used.

## 4. Conclusions

This work used the solid dispersion method to produce curcumin-loaded microparticles with Pickering stabilizing action, directed to the development of novel and promising food formulations. Using k-carrageenan as the matrix polymer, two formulations using different curcumin contents were prepared, KC1 and KC2, showing low particle size in volume (D_50_ of 2.39 and 3.29 µm, respectively), which is desirable for their role as Pickering stabilizers. Both particles evidenced a hydrophilic character with a water contact angle of 34.97° and 23.78° for KC1 and KC2, respectively, showing their suitability to stabilize O/W emulsions. The particles were employed in the preparation of Pickering emulsions holding different oil volume fractions and particle contents (KC1 φ 0.4 (4.7%); KC2 φ 0.4 (4.7%) and KC2 φ 0.6 (4.0%)), showing gel-like aspects, indicative of high resistance to instability phenomena. This fact was confirmed by CI and optical microscopy analysis, where the systems remained stable over 28 days under ambient conditions, manifesting a morphology characterized by uniform and round-shape droplets. Only for the KC2 φ 0.6 (4.0%) formulation was a small oil accumulation at the top of the emulsion detected for times over 14 days.

The comparison of the produced emulsions with commercial mayonnaises (traditional and light) evidenced their promising characteristics as mayonnaise-like food sauce substitutes with the advantages of holding a low-fat content. Moreover, the three produced emulsions showed an acidic pH, a considerably improved oxidative stability, and an attractive bright yellow color. Among them, the sample KC1 φ 0.4 (4.7%) showed similar textural properties to light mayonnaise, positioning this formulation as a promising system. It is based on natural products, has fat-reduced properties, and extra benefits imparted by curcumin and virgin olive oil. The next steps on this ongoing work will consider sensory assessment aiming at evaluating consumers’ acceptance.

Overall, the developed solution offers the potential to be extended to other hydrophobic natural colorants ensuring the development of products comprising a wide range of colors. Pickering emulsion-based products can be also produced with different viscosities and textures, highlighting the versatility of this approach.

## Figures and Tables

**Table 1:** Particle size in volume and number expressed as D_10_, D_50_, and D_90_.

Sample	Particle Size in Volume			Particle Size in Number		
	D_10_ (µm)	D_50_ (µm)	D_90_ (µm)	D_10_ (µm)	D_50_ (µm)	D_90_ (µm)
KC1	0.56 ± 0.00	3.29 ± 0.02	164 ± 1.69	0.37 ± 0.00	0.48 ± 0.00	0.69 ± 0.00
KC2	0.53 ± 0.00	2.39 ± 0.01	70.2 ± 2.26	0.37 ± 0.00	0.48 ± 0.00	0.69 ± 0.00

**Table 2:** L*, a* and b* color parameters of the produced particles.

Sample	L*	a*	b*	RGB Color
KC1	85.57 ± 0.02	6.69 ± 0.02	93.95 ± 0.02	
KC2	88.21 ± 0.03	0.77 ± 0.02	97.70 ± 0.00	

**Table 3:** Droplet size of the produced Pickering emulsions (KC1 φ 0.4 (4.7%), KC2 φ 0.4 (4.7%), KC2 φ 0.6 (4.0%)) over time (t0–t28 (days)).

	Average Size of Droplets (µm)		
Time (Days)	KC1 φ 0.4 (4.7%)	KC2 φ 0.4 (4.7%)	KC2 φ 0.6 (4.0%)
t0	5.93 ± 2.66	6.56 ± 3.04	10.73 ± 4.80
t7	6.03 ± 2.77	6.66 ± 3.51	14.22 ± 6.31
t14	6.18 ± 1.61	7.34 ± 3.54	20.06 ± 5.78
t21	6.68 ± 2.00	8.06 ± 4.02	29.19 ± 15.15
t28	7.68 ± 1.92	8.95 ± 5.32	35.10 ± 14.69

**Table 4:** Average values for the color parameters (L*, a* and b*) of the produced Pickering emulsions and commercial products (traditional and light mayonnaise).

Sample	L*	a*	b*	RGB Color	pH	Oxidative Stability (h)
KC1 φ 0.4 (4.7%)	80.13	1.74	72.87		4.62 ± 0.02	24.28
KC2 φ 0.4 (4.7%)	84.42	−6.40	71.58		4.55 ± 0.04	27.98
KC2 φ 0.6 (4.0%)	81.18	−15.61	70.42		3.70 ± 0.03	20.32
Traditional mayonnaise	93.02	−1.61	18.15		3.61 ± 0.02	0.34
Light mayonnaise	92.66	−2.52	25.18		3.42 ± 0.07	0.72

**Table 5:** Textural properties of the reference food matrices and the Pickering emulsions over time.

Sample	Time (min)	Firmness (g)	Consistency (g·s)	Cohesiveness (g)	Work of Cohesion (g·s)
Traditional mayonnaise	0	395.37± 0.94 ^a^	946.25± 61.99 ^a^	(−) 219.57 ± 19.38 ^bc^	(−) 3.52 ± 0.96 ^a^
	10	369.8 ± 44.83 ^a^	563.88 ± 489.83 ^a^	(−) 239.21 ± 4.79 ^c^	(−) 42.39 ± 38.64 ^ab^
Light mayonnaise	0	374.30 ± 13.20 ^a^	865.54 ± 48.07 ^a^	(−) 204.30 ± 0.26 ^bc^	(−) 1052.84 ± 315.56 ^c^
	10	361.70 ± 7.24 ^ab^	762.67 ± 184.49 ^a^	(−) 211.01 ± 9.92 ^bc^	(−) 773.27 ± 7.06 ^abc^
KC1 φ 0.4 (4.7%)	0	359.29 ± 6.41 ^ab^	885.44 ± 15.89 ^a^	(−) 204.04 ± 2.94 ^bc^	(−) 1081.38 ± 289.98 ^c^
	10	332.94 ± 0.34 ^abc^	787.70 ± 34.28 ^a^	(−) 196.46 ± 1.70 ^abc^	(−) 817.55 ± 50.21 ^bc^
KC2 φ 0.4 (4.7%)	0	273.33 ± 1.06 ^bcd^	643.29 ± 26.07 ^a^	(−) 180.06 ± 0.68 ^ab^	(−) 329.26 ± 78.99 ^abc^
	10	260.40± 8.48 ^cd^	581.90 ± 87.06 ^a^	(−) 177.87 ± 8.97 ^ab^	(−) 324.47 ± 62.52 ^abc^
KC2 φ 0.6 (4.0%)	0	189.67 ± 15.87 ^d^	427.78 ± 0.88 ^a^	(−) 148.62± 8.22 ^a^	(−) 151.73 ± 3.05 ^ab^
	10	184.77 ± 13.91 ^d^	465.50 ± 7.71 ^a^	(−) 150.47 ± 9.39 ^a^	(−) 170.48 ± 0.17 ^ab^

Results are presented as average ± standard deviation. Different letters correspond to significant differences (*p*-value < 0.05).

**Table 6:** Formulation of the particles produced by solid dispersion.

Sample	Curcumin (% *w*/*w*, Polymer-Basis)	pH	Tween 80 (% *w*/*w*, Polymer-Basis)
KC1	15.00	4.50	15.00
KC2	8.88	3.58	24.19

**Table 7:** Formulation of the produced Pickering emulsions.

Sample	Particle Type	PC (% *w*/*w*)	φ (*v*/*v*)
KC1 φ 0.4 (4.7%)	KC1	4.7	0.4
KC2 φ 0.4 (4.7%)	KC2	4.7	0.4
KC2 φ 0.6 (4.0%)	KC2	4.0	0.6

## Data Availability

Data sharing not applicable.

## Sample Availability

Samples of the compounds are not available from the authors.
